# Efficient Isolation of Mouse Liver NKT Cells by Perfusion

**DOI:** 10.1371/journal.pone.0010288

**Published:** 2010-04-21

**Authors:** Xianfeng Fang, Peishuang Du, Yang Liu, Jie Tang

**Affiliations:** 1 Center for Infection and Immunity, Institute of Biophysics, Chinese Academy of Science, Beijing, China; 2 Graduate University of Chinese Academy of Sciences, Beijing, China; 3 Departments of Surgery, Internal Medicine and Pathology, University of Michigan at Ann Arbor, Ann Arbor, Michigan, United States of America; New York University, United States of America

## Abstract

**Background:**

NKT cell is a population of unconventional T cells that mediate both innate and adaptive T cell responses. Since NKT cells are most abundant in the liver, much of NKT biology has been learnt from studies of NKT cells isolated from liver. This is a cumbersome procedure with variations in cell yield.

**Results:**

Based on recent evidence that NKT cells reside in liver vascular compartment, we developed a simple method to isolate NKT cells by perfusion with PBS-containing 10 mM of EDTA. The number and cell surface phenotype of liver NKT cells recovered by perfusion and by the traditional method were comparable. The yield of other lymphocytes was also comparable.

**Conclusion/Significance:**

Our data demonstrated that liver lymphocytes can be efficiently isolated by simple perfusion. These data provide a convenient method to isolate liver lymphocyte while preserving liver tissue for other analysis.

## Introduction

NKT cells are important regulators and effectors in both innate and adaptive immunity. Mouse NKT cell were broadly defined as a subpopulation of T cells that share some characteristics with NK cell, particularly expression of NK1.1 [1]. Mouse NKT cells have been divided into two subsets. Type I NKT cells have a largely invariable T cell receptor a-chain (Vá14/Já18) and are thus called iNKT. Type II NKT cells refer to all other CD1d-dependent T cells [2]. NKT cells have been implicated in immune responses against infectious agents and tumors, and in regulating a wide variety of autoimmune and inflammatory diseases [1].

Although NKT cell can be detected wherever conventional T cell are found, the highest NKT cell/T cell ratio is found in the liver [3]. Thus, whereas mouse NKT cells represent about 0.5% of the T cell population in the blood, peripheral lymph node, and spleens, almost 30% of the T cell in the liver have the NKT phenotype [3]. Due to the abundance of NKT cells in mouse liver, liver NKT cells are routinely isolated to study the biology of NKT cells. The procedure of isolating NKT cells typically involves perfusion, mechanical segregation and gradient centrifugation [4]. Recently, it was realized that NKT cell, like others immune cells (such as conventional T cells, NK cells, and dendritic cells), is localized in the liver sinusoids [5]. Since the NKT cells are largely vascular, we tested the possibility if they can be isolated simply by perfusion with cation chelator-containing buffer.

## Materials and Methods

### Mice

The handling of mice and experimental procedures were approved by the Animal Welfare and Research Ethics Committee of the Institute of Biophysics, Chinese Academy of Sciences. Protocol No. IBP-2007129. Six- to 12-week-old C57BL/6 mice were maintained under specific pathogen-free conditions and used throughout these experiments.

### Antibodies and tetramers

The monoclonal antibodies CD3-FITC or CD3-APC (clone 145-2C11), NK1.1-PE or NK1.1-PerCp (clone PK136), CD4-FITC (clone GK1.5) were purchased from eBioscience (San Diego, CA). The PE-CD1d-GSL tetramer was provided by the tetramer facility at the National Institutes of Health.

### Other materials

The closed catheter system (BD Intima IITM) purchased from Becton Dickinson (Suzhou, China).

### Preparation of cell suspension

Liver lymphocytes were prepared with the traditional method according to the methods of Matsuda with minor modified [4]. In briefly, after perfusion with 3 ml PBS via portal vein, mouse liver fragments were pressed through 70-ìm cell strainer (Becton Dickinson). Total liver cells were then resuspended in a 40% isotonic Percoll solution (Amersham Pharmacia Biotech) underlaid with a 60% isotonic Percoll solution. After centrifugation for 25 minutes at 800 g, mononuclear cells were isolated at the 40/60% interface. The cells were washed once with DMEM (Gibco, BRL) supplemented with 5% FBS (Hyclone).

### Detailed protocol of isolating liver lymphocytes by perfusion

1. Mouse abdomen was opened immediately after cervical dislocation. Portal vein were cannulated with a 24 G Intravenous Catheters and fixed the catheters with an artery clamp.
2. Inject 1 ml solution containing10 mM EDTA and 100 U heparin in PBS via the catheters.
3. Ligate both of the right and left renal veins with suture.
4. Cannulate the inferior vena cava with an 18 G needle and tightly lie the need to the vein with suture.
5. Open the thorax and ligate the superior vena cava with an artery clamp.
6. Perfuse the liver with 3 ml PBS via the catheters.
7. Perfuse the liver with 20 ml or more 10 mM EDTA/PBS with 4 ml/min rate and collect the flow out with a 100 mm dish under the 18 G needle.
8. Transfer the flow out to the centrifuge tube and centrifuge 5 minutes at 400 g.
9. Resuspend the cell pellet with the 1 ml ACK Lysis buffer(0.15 M NH4Cl, 10 mM KHCO3, 0.1 mM Na2EDT, adjust PH to 7.2–7.4).
10. Wash the cell once with DMEM supplemented with 5% FBS. Count cell with hematocytometer.

### FACS analysis

Cell preparations were stained with the antibody cocktail include CD3-FITC, CD4-APC, NK1.1-PerCP and CD1d-aGalCer tetramer-PE, incubation was performed at 4°C for 30 minutes in the dark in 100 µl staining buffer (2% FBS and 0.1% sodium azide in PBS). Samples were then washed and examined by flow cytometer with CellQuest software (BD FACSCalibur, Becton Dickinson and Company) and the data were analyzed with flowjo 5.72.

### Statistics

Prism software (GraphPad) was used for all statistical analyses. Unpaired Student t tests Were used to compare the experimental groups. A P value of less than 0.05 was accepted as being significant.

## Results and Discussion

**Figure 1:**
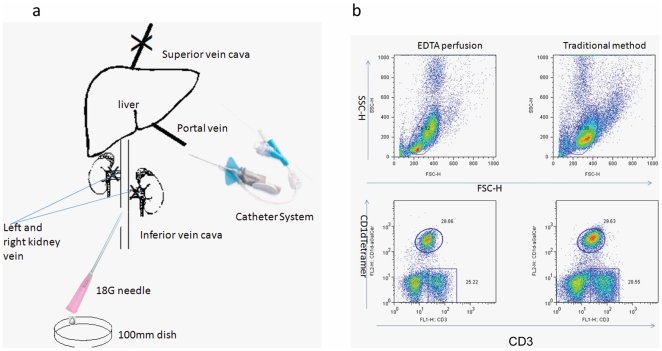
A new method for NKT cell isolation from liver. (a) A diagram of liver perfusion. (b) The representative FACS profile of cells isolated by either perfusion or by the traditional methods.

According to the blood flow in the liver, we design the perfusion procedure as diagramed in Fig. 1a. Based on the profiles of CD3 expression and CD1d-GSL tetramer, it is clear that the frequency of iNKT is very similar in the flow out of EDTA perfusion and the cells prepared by traditional methods.

**Figure 2:**
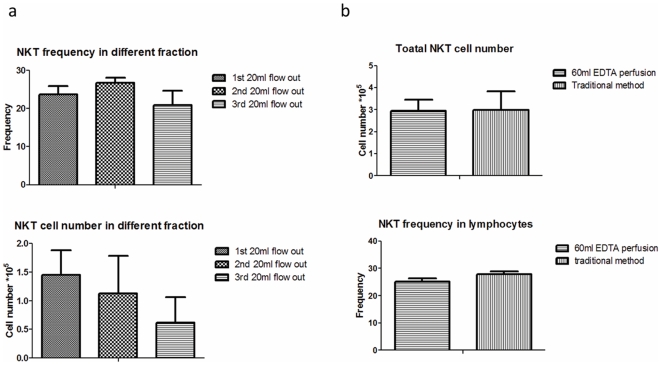
Yields and frequency of NKT cells isolated by perfusion vs. the traditional methods. (a). Livers were perfused with 60 ml 10 mM EDTA/PBS and the three fractions of 20 ml flowouts were collected. Both the frequency (upper left panel) and the total cell number (lower left panel) of NKT cells in the flowouts were compared. (b) The frequencies (upper right panel) and cell numbers (lower right panel) of NKT cells isolated by perfusion with 60 ml EDTA buffer were comparable to those isolated by traditional methods. Data shown are means and S.D., n = 3. The experiment repeated 3 times.

**Figure 3:**
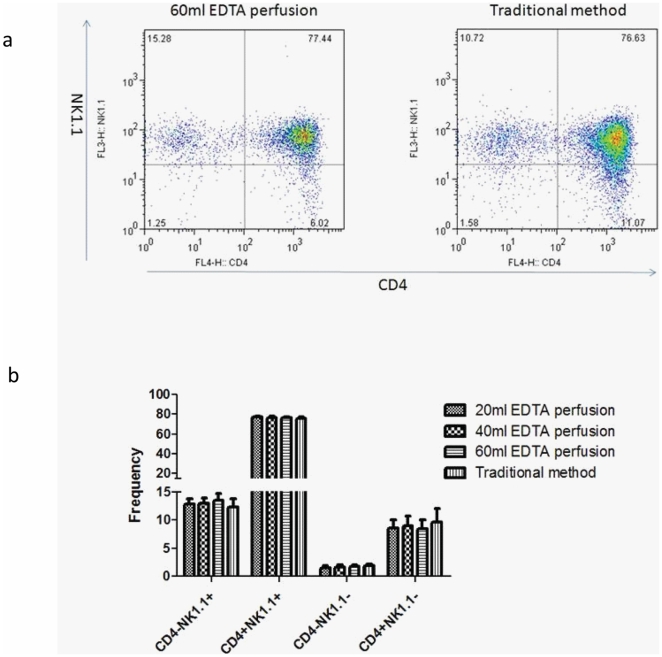
Similar phenotypes of NKT cells isolated by two methods. (a) representative FACS profile of NKT cell isolated with two methods. (b) Summary data. Data shown are means and S.D., n = 3.

To determine the amounts of perfusion needed to recover NKT cells, we perfuse the liver with 60 ml EDTA buffer and collect the first, the second and the third 20 ml flow. The frequency and the total NKT cell number were determined by flow cytometry. As shown in Fig. 2a upper panel, the frequency of iNKT in the flow out are unchanged in the three fractions, which indicated that iNKT cells are not more adherent than other cell types. However, the yield of iNKT cells reduced in each round. When the fractions are combined, a comparable number of iNKT cells were recovered by either perfusion or by the traditional methods (Fig 2b). Since iNKT cells can be divided into different subsets based on expression of CD4 and NK1.1, we compared the phenotype of the iNKT cells. As shown in Fig. 3, the gated tetramer+ NKT cells prepared by the two different methods are also comparable.

**Figure 4:**
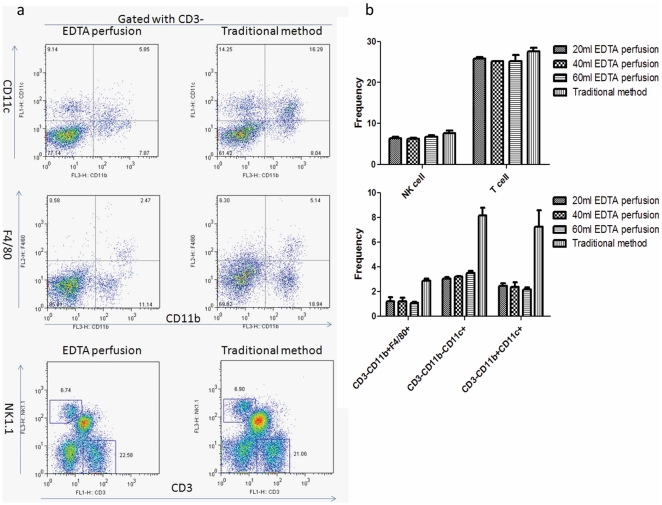
Characterization of cellular composition of liver leukocytes isolated by the two methods. (a) Representative FACS profile of NK cells, T cells, macrophages cell and dendritic cells of liver lymphocytes isolated with two methods. (b) Summary data. Data shown are means and S.D., n = 3. These data have been repeated twice.

In order to investigate whether others immune cell can be isolated with the new method, we analyzed the frequency T cell, NK cell, macrophages and dendritic cell in the flow out. Total T cell were gated with CD3^+^ and the NK cell gated with CD3^−^NK1.1^+^ in the experiment. Macrophages were defined as CD3^−^CD11b^+^F4/80^+^ while the liver dendritic cells were marked as CD3^−^CD11c^+^CD11b^+^ or CD3^−^CD11c^+^CD11b^−^. We found that both T cell and NK cell can be isolated with the new method. However, only 40–50% of macrophages can be isolated by perfusion (Fig 4b). This is expected as many liver macrophages reside within liver parenchyma.

Taken together, we devised a new method for liver NKT cell isolation based on the observation that NKT cells are largely vascular [4]. Compared with the traditional method, the new method has at least two merits. First, the new method is less labor-intensive and time consuming. Second, the new method allows isolation of NKT cells while preserving the liver tissue for other analysis.

## References

[1] Bendelac A, Savage PB, Teyton L (2007). The biology of NKT cells.. Annu Rev Immunol.

[2] Godfrey DI, MacDonald HR, Kronenberg M, Smyth MJ, Van Kaer L (2004). NKT cells: what's in a name?. Nat Rev Immunol.

[3] Eberl G, Lees R, Smiley ST, Taniguchi M, Grusby MJ (1999). Tissue-specific segregation of CD1d-dependent and CD1d-independent NK T cells.. J Immunol.

[4] Matsuda JL, Naidenko OV, Gapin L, Nakayama T, Taniguchi M (2000). Tracking the response of natural killer T cells to a glycolipid antigen using CD1d tetramers.. J Exp Med.

[5] Geissmann F, Cameron TO, Sidobre S, Manlongat N, Kronenberg M (2005). Intravascular immune surveillance by CXCR6+ NKT cells patrolling liver sinusoids.. PLoS Biol.

